# Utilizing Gold Nanoparticles as Prospective Radiosensitizers in 3D Radioresistant Pancreatic Co-Culture Model

**DOI:** 10.3390/ijms241512523

**Published:** 2023-08-07

**Authors:** Abdulaziz Alhussan, Nolan Jackson, Reinali Calisin, Jessica Morgan, Wayne Beckham, Devika B. Chithrani

**Affiliations:** 1 Department of Physics and Astronomy, University of Victoria, Victoria, BC V8P 5C2, Canada; alhussan@uvic.ca (A.A.); nolanjackson12@uvic.ca (N.J.); rcalisin@uvic.ca (R.C.); wbeckham@bccancer.bc.ca (W.B.); 2 Department of Biochemistry and Microbiology, University of Victoria, Victoria, BC V8P 5C2, Canada; jemorgan@bccrc.ca; 3 Trev and Joyce Deeley Research Centre, British Columbia Cancer-Victoria, Victoria, BC V8R 6V5, Canada; 4 Radiation Oncology, British Columbia Cancer-Victoria, Victoria, BC V8R 6V5, Canada; 5 Centre for Advanced Materials and Related Technologies, Department of Chemistry, University of Victoria, Victoria, BC V8P 5C2, Canada; 6 Department of Medical Sciences, University of Victoria, Victoria, BC V8P 5C2, Canada; 7 Department of Computer Science, Mathematics, Physics and Statistics, Okanagan Campus, University of British Columbia, Kelowna, BC V1V 1V7, Canada

**Keywords:** gold nanoparticles, radiosensitizers, 3D spheroids, co-culture, pancreatic cancer

## Abstract

Pancreatic cancer stands among the deadliest forms of cancer, and the existing treatments fall short of providing adequate efficacy. Novel and more effective treatment approaches are urgently required to address this critical medical challenge. In this study, we aimed to evaluate the anti-cancer efficacy of gold nanoparticles (GNPs) in combination with radiotherapy (RT). A 3D pancreatic cancer co-culture spheroid model of MIA PaCa-2 cancer cells and patient-derived cancer-associated fibroblasts (CAF-98) was used. The spheroids were treated with GNPs (7.5 μg/mL) and 2 Gy of RT. The spheroids’ cell viability was assessed through the CellTiter-Glo 3D assay, and an immunofluorescence assay was used to assess the DNA DSBs via the expression of the DNA damage marker 53BP1. Co-culture samples showed a 10.8% (*p* < 0.05) increase in proliferation and a 13.0% (*p* < 0.05) decrease in DNA DSB when compared to monoculture samples, However, they displayed a 175% (*p* < 0.001) increase in GNPs uptake when compared to monoculture spheroids. Using GNPs/RT, we were able to show a significant reduction of 6.2% (*p* < 0.05) in spheroid size and an increase of 14.3% (*p* < 0.05) in DNA DSB damage in co-culture samples. The combination of GNPs with RT demonstrated remarkable radiosensitization effects, representing a promising approach to enhance cancer treatment efficacy. These effects were particularly noteworthy in the more treatment-resistant co-culture spheroid model.

## 1. Introduction

Pancreatic cancer is one of the deadliest forms of cancer. According to the American Cancer Society, pancreatic cancer is the third leading cause of cancer-related deaths in the United States [1]. The 5-year survival rate for pancreatic cancer is only around 10% [2]. It is estimated that in 2021, there will be 60,430 new cases of pancreatic cancer in the United States and 48,220 deaths from the disease [3]. One of the major shortcomings of current treatments for pancreatic cancer is that it is often diagnosed at an advanced stage when cancer has already spread beyond the pancreas [4]. This makes it more difficult to treat and is one of the reasons why the survival rate is so low. Surgery is often the best option for treating pancreatic cancer, but many patients are ineligible due to the advanced stage of their disease [2]. Chemotherapy is also commonly used, but this has significant side effects and is often not very effective due to the complex tumor microenvironment [5]. Despite immunotherapy success in many other cancers, there has been little progress in incorporating immunotherapy into the treatment approach for pancreatic cancer. Clearly newer and more innovative treatment options are needed for this cancer [6].

The use of gold nanoparticles (GNPs) in radiotherapy (RT) is an emerging field that has shown great potential to improve the effectiveness of radiation treatment for cancer [7]. GNPs are nanometer-sized gold particles, typically between 1–100 nm in diameter, that can be easily engineered to bind selectively to cancer cells in the body [8]. When GNPs are exposed to radiation, they can absorb the energy from the radiation and locally deposit it [9]. This localized energy deposition can damage cancer cells more effectively while sparing healthy surrounding tissue [10]. One of the most promising applications of GNPs in RT is as a radiosensitizer for enhancing the effects of clinical radiation doses [11]. By selectively delivering GNPs to tumor sites, radiation doses can be increased to the cancer cells while minimizing damage to normal cells [12]. Recent studies have shown promising results in using GNPs with RT for various types of cancer, such as prostate, breast, and lung cancer [13].

**Figure 1:**
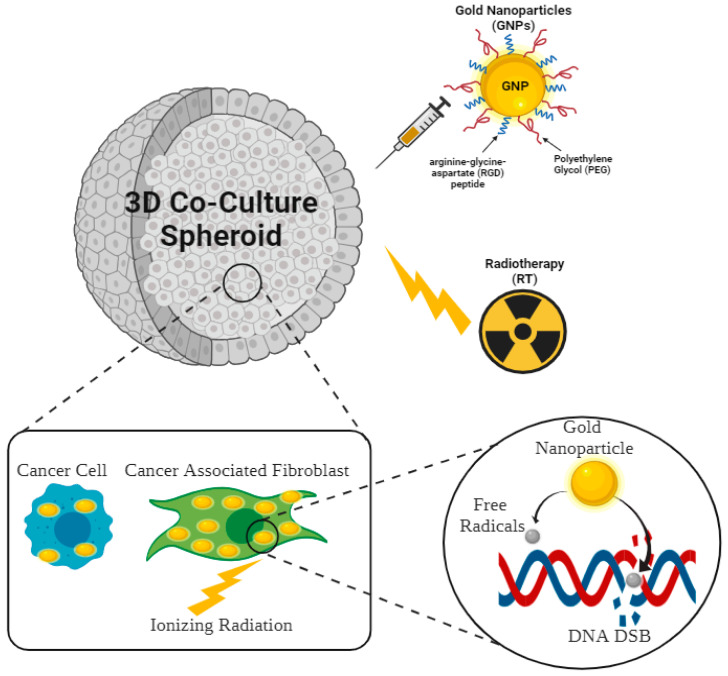
Schematic showing the combined modality of gold nanoparticles (GNPs) functionalized with polyethylene glycol (PEG) and arginine-glycine-aspartate (RGD) peptide, and radiotherapy (RT) in a 3D co-culture model of cancer cells and cancer associated fibroblasts (CAFs). The subset shows the mechanism of GNPs radiosensitization. GNPs absorb the energy of the incident photon and deposit it in the cell causing the production of free radicals in the vicinity of the DNA leading to DNA DSB.

To best examine the combined effects of GNPs and RT in vitro, it is important to understand the tumor microenvironment (TME). TME refers to the surrounding environment in which a tumor exists, including the cells, extracellular matrix, and signaling molecules [14]. The TME is complex and dynamic, consisting of both pro-tumorigenic and anti-tumorigenic factors that influence tumor growth and progression [15]. Cancer-associated fibroblasts (CAFs) are an important part of the TME. They are activated fibroblasts that produce a range of proteins and growth factors that can promote tumor growth and invasion [16]. CAFs can also remodel the extracellular matrix, promoting tumor cell migration and invasion; thus, CAFs can also contribute to tumor progression [17]. Unlike the traditional 2D cell culture model, a 3D spheroid mimics the conditions of the TME more accurately, since the cells are grown together in 3D arrangements similar to that found in tumors [18]. The main advantages of using a 3D spheroid model to test cancer drugs in vitro include increased physiological similarity to tumors when compared to traditional 2D cell cultures, allowing for more accurate testing of drug efficacy, and improved in vivo predictive outcomes when compared to 2D cell culture models [19]. However, it is important to note that the majority of studies in the literature that utilize GNPs as radiosensitizers tend to focus on culture models that do not include CAFs. Therefore, in this paper, we recreated some of the complexities of the TME using an in vitro 3D spheroid model made of MIA PaCa-2 and patient-derived CAFs of pancreatic origin. Subsequently, we evaluated the efficacy of combining clinically relevant doses of GNPs (7.5 μg/mL) and RT (2 Gy), a strategy that had not been explored previously in the presented 3D co-culture model (Figure 1). The goal is to address the following questions:

1. Is there an improvement in RT with the addition of GNPs as radiosensitizers when compared to RT alone in monoculture and in co-culture in vitro 3D models?
2. Does the inclusion of CAFs in cancer cells in vitro increase resistance to the proposed treatments?

## 2. Results and Discussions

### 2.1. Monoculture and Co-Culture 3D Spheroids

**Figure 2:**
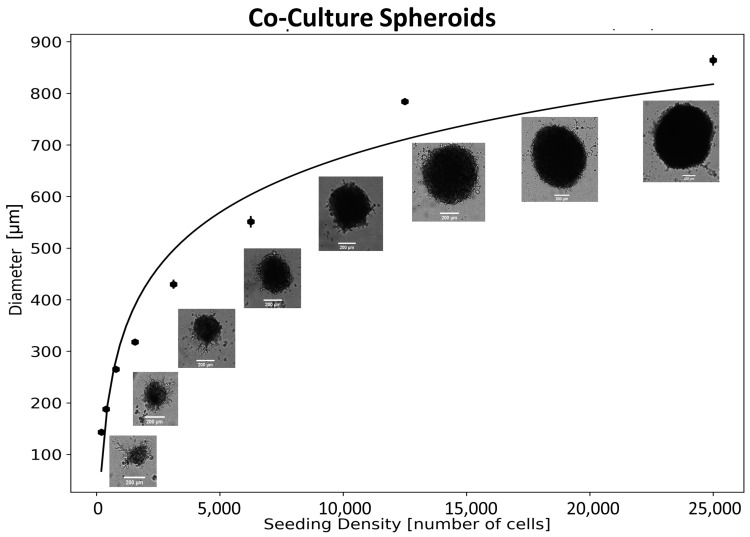
Characterizing pancreatic cancer 3D spheroid size. The size of the spheroids for a 5:1 ratio of patient-derived CAF-98 to MIA PaCa-2 co-culture under different initial cell count seeding, error bars showing standard error (S.E.). The scale bar is 200 µm.

Pancreatic cancer 3D spheroidal models were formed using MIA PaCa-2 and patient-derived pancreatic cancer-associated fibroblasts, CAF-98, and were grown in vitro in ultra-low attachment 96-well microplates. The 3D monocultures were formed using only the MIA PaCa-2 cell line, while 3D co-culture spheroids were formed using a 5:1 ratio of CAF98 to MIA PaCa-2. Our previous study has shown that this ratio resulted in increased resistance in 2D co-culture models [20]. The number of cancer cells initially seeded determines the approximate diameter of the spheroid 72 h post-seeding. This is supported by brightfield images of different spheroid sizes in 96-well microplates 72 h post-seeding as shown in Figure 2 (co-culture) and Figure S1 (monoculture). An approximate size of 300–400 µm was used for all experiments. This corresponds to a seeding density of approximately 6000 cells for the monoculture and approximately 1800 cells for the co-culture. These spheroid sizes were used because the distance between capillaries in solid tumors is around 100–200 μm, representing roughly the radius of our spheroids [21]. This would not introduce hypoxia, which could be a confounding factor that is difficult to account for [22]. Co-culture models that include cancer-associated fibroblasts (CAFs) are better suited for studying pancreatic cancer in vitro as they more accurately represent the complex and heterogeneous TME [23]. The interaction between pancreatic cancer cells and CAFs is known to play a crucial role in the proliferation, invasion, and chemoresistance of pancreatic cancer through the secretion of growth factors and extracellular matrix components [24]. By co-culturing pancreatic cancer cells and CAFs, we can mimic the pancreatic TME in order to study the effectiveness of our treatment modality more accurately [25]. These 3D spheroidal models would allow for the study of pancreatic cancer behavior in a more physiologically relevant environment when compared to traditional 2D monolayer cell cultures [26]. Additionally, these models can help to reduce the use of animal models and improve translation into clinical settings [25,26,27]. The in vitro 3D co-culture spheroids model, while valuable, has certain limitations that need to be acknowledged. The 3D co-culture spheroid model is still an artificial representation of the complex TME found in the highly heterogeneous in vivo environment. It does not fully capture the interactions and dynamics of different cell types, extracellular matrix components, and immune responses present in real tumors [26]. Additionally, these spheroids lack a functional vasculature system, which limits the diffusion of nutrients and oxygen within the core of the spheroid, especially for larger spheroids [27]. This can affect the growth and viability of cells, potentially leading to differences in drug responses when compared to the in vivo situation. Despite these limitations, the 3D co-culture spheroid model remains a valuable tool for studying tumor biology, drug responses, and interactions between different cell types in a controlled and reproducible environment. 

### 2.2. Gold Nanoparticles Uptake in 3D Monoculture and 3D Co-Culture

GNPs that are approximately 13 nm in diameter were used for all experiments. These small sizes have several advantages that make them attractive for various biomedical applications, including both therapy and imaging. These advantages include better surface functionalization due to having a high surface area to volume ratio, higher tumor accumulation due to the enhanced permeability and retention (EPR) effect, and higher overall versatility, which allows them to be used in various imaging applications [28]. All cells were treated with GNPs at a concentration of 7.5 μg/mL. These concentrations are considerably lower than concentrations that would cause toxicity to cells [29]. This is important to facilitate the transition into clinical trials in the future. The GNPs were functionalized with polyethylene glycol (PEG) and the arginine-glycine-aspartate (RGD) peptide to enhance their biocompatibility and provide properties for targeting cancer cells and CAFs. PEGylation of GNPs can enhance their biocompatibility by reducing their toxicity and immunogenicity [30]. This increases their circulation time in the bloodstream and reduces clearance by the reticuloendothelial system [31]. PEGylation can also increase the stability of GNPs by preventing their aggregation and reducing non-specific binding to cells and tissues [32]. Functionalization of GNPs with the RGD peptide enables targeted delivery to cells that express integrin receptors [33]. The RGD peptide specifically binds to αvβ6 and αvβ3 integrin receptors, which are overexpressed on pancreatic cancer cells and CAFs [34]. As demonstrated in our previous research, normal fibroblasts lacking αvβ3 integrin receptors exhibited less than 3% uptake in GNPs compared to CAFs [35]. When GNPs are functionalized with RGD, the RGD peptide acts as a targeting ligand, allowing the nanoparticles to selectively bind to these cells. Upon binding to the integrin receptors, the GNPs can be internalized into the cells by receptor-mediated endocytosis [9]. The use of RGD-functionalized GNPs can improve the efficacy of cancer therapies and minimize off-target effects. The stability of the gold nanoparticle complexes is shown in Figure S2.

**Figure 3:**
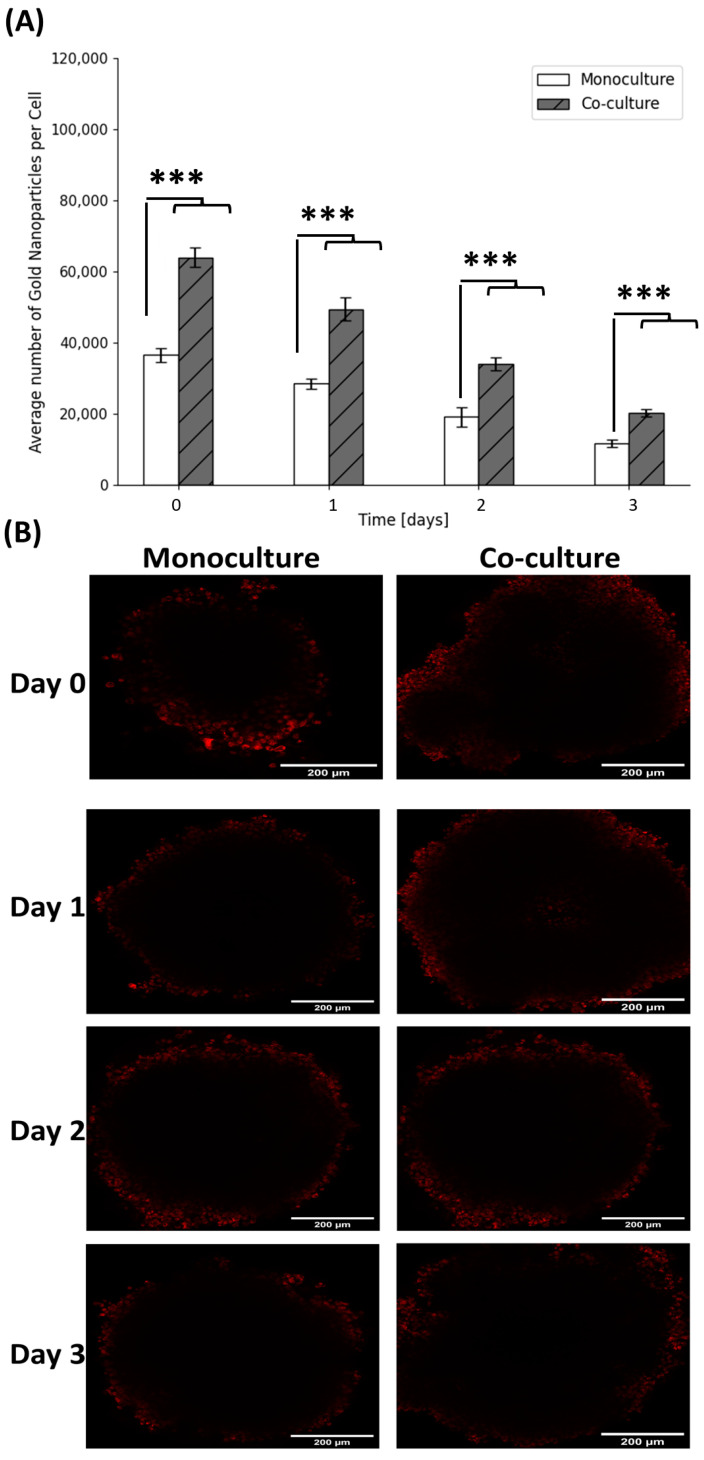
Gold nanoparticle (GNP) uptake and retention in pancreatic cancer spheroids. (**A**) Quantification of uptake of 7.5 µg/mL of GNPs into MIA PaCa-2 monoculture spheroids and CAF-98 to MIA PaCa-2 (5:1) co-culture spheroids as measured using ICP-MS, error bars showing standard error (S.E.). *** indicates *p* < 0.001. (**B**) Confocal Images of GNPs’, in red, uptake, and retention over 3 days in MIA PaCa-2 monoculture spheroids and CAF98 to MIA PaCa-2 (5:1) co-culture spheroids. Scale bar: 200 µm.

After an incubation period of 24 h with GNPs, the media was changed to mimic the loss of GNPs supply in the body following a single injection. The samples were then processed and the amount of gold in each cell was calculated as described in Section 3.5. The amount of gold in monoculture cells and co-culture cells over time is shown in Figure 3A. There was an increase of about 175% in the uptake of gold in the co-culture samples when compared to monoculture at day 0 and on the 3 consecutive days. This increase is attributed to the effect of CAFs in the co-culture. CAFs are, on average, 4 times larger than MIA PaCa-2 and can internalize 2–3 times the number of GNPs compared to cancer cells [20]. If we factor in the size of the cells, MIA PaCa-2 takes up approximately twice the amount of gold per unit volume relative to CAFs. It is also important to note that the difference in the percentage of GNPs retained in cells in monoculture vs. co-culture was insignificant. After the uptake of gold, the co-culture spheroids demonstrated a retention rate of 77.4%, 53.2%, and 31.7% on the three consecutive days, respectively. In comparison, the monoculture spheroids exhibited retention rates of 77.8%, 52.5%, and 32.1% during the same period, respectively. These results are supported qualitatively by confocal images (Figure 3B) of the spheroids over a 3-day period, visually showing higher of GNPs in cells at day 0 compared to day 3 in co-culture compared to monoculture.

### 2.3. Gold Nanoparticles and Radiotherapy in 3D Spheroids

**Figure 4:**
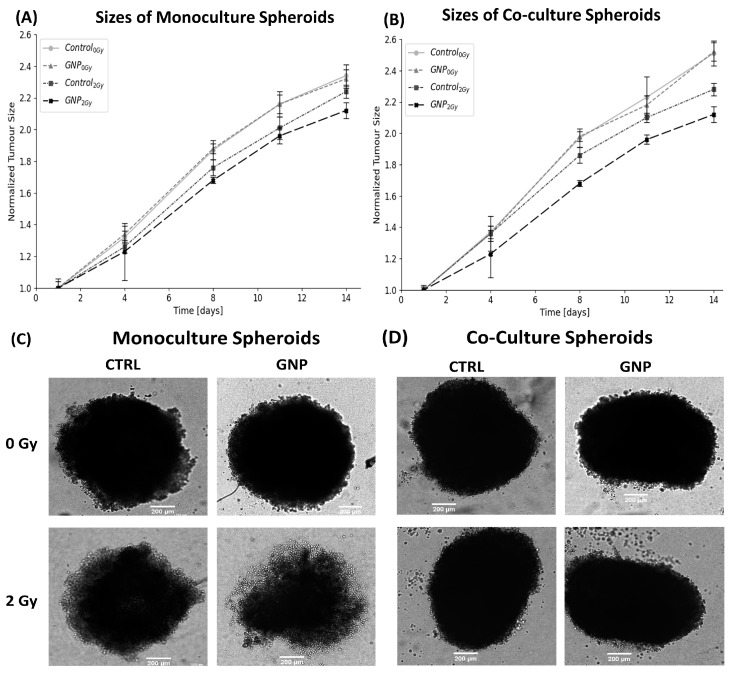
Monoculture vs. co-culture spheroids sizes post-treatment with RT/GNP. (**A**,**B**) Normalized monoculture (**A**) and co-culture (**B**) spheroids size over 14 days post-treatment, error bars showing standard error (S.E.). (**C**,**D**) Bright-Field images of monoculture spheroids (**C**) and co-culture spheroids (**D**) taken 14 days post-treatment. Scale bar: 200 µm.

After an incubation period of 24 h with GNPs, the media was changed, and the samples were irradiated with a single 2 Gy dose. Figure 4A,B displays the relative change in diameter of monoculture and co-culture spheroids over 14 days following treatment with radiation. Both monoculture (Figure 4A) and co-culture (Figure 4B) spheroids behaved as expected. GNPs for non-irradiated spheroids presented no effects on spheroid size, showing no toxicity at the concentration used. On the other hand, the use of GNPs with radiation resulted in 5.5% and 6.2% in tumor size shrinkage for monoculture and co-culture, respectively, compared to using radiation alone. These results are visualized using brightfield images of the spheroids 14 days post-treatment (Figure 4C,D). To confirm our previous results, 3D viability assays were conducted (Figure S3). The results agree with the tumor size results (Figure 4). With radiation treatment, the use of GNPs displayed a significant reduction of 15.1% and 10.3% in cell proliferation in both monoculture and co-culture, receptively, compared to using radiation alone (Figure S3A). With no radiation, GNPs had no effect on cell proliferation in either monoculture or co-culture (Figure S3B). Additionally, it is noteworthy that the co-culture cells displayed a proliferation increase of 4.8% and 10.8% compared to monoculture cells in the irradiated samples and the irradiated samples with GNPs, respectively.

The expected results are credited to the radiosensitization effects of GNPs. When exposed to RT, GNPs can absorb the radiation energy, leading to the emission of short-ranged electrons [13]. These electrons deposit their energy locally within and around the internalized nanoparticles, leading to the ionization of water molecules inside cancer cells. This ionization generates free radicals and reactive oxygen species (ROS) [13]. The interaction of electrons and ROS with DNA molecules can lead to the formation of DNA DSB. The cumulative effect of radiation-induced damage, particularly DNA DSBs, can trigger cell death pathways, leading to the elimination of cancer cells [13]. The observed increase in the proliferation of co-culture spheroids compared to monoculture ones might be attributed to the higher expression of integrin receptors on pancreatic cancer cells and CAFs. This overexpression is known to contribute to the aggressive behavior of pancreatic cancer cells and their resistance to therapy [36]. It is thought to contribute to the desmoplastic response seen in pancreatic cancer, which is characterized by the deposition of fibrous tissue and the formation of a dense extracellular matrix that can impede drug delivery [36]. Several studies have investigated the role of specific integrin receptors in pancreatic cancer progression and metastasis. For example, αvβ6 integrin has been shown to be overexpressed on pancreatic cancer cells and to play a role in tumor invasion and metastasis [37]. Inhibition of αvβ6 integrin has been shown to decrease tumor growth and metastasis in pre-clinical models of pancreatic cancer [37]. Similarly, αvβ3 integrin has also been found to be overexpressed on pancreatic cancer cells and to contribute to tumor invasion and angiogenesis [38]. Inhibition of αvβ3 integrin has been shown to reduce tumor growth and angiogenesis in xenograft models of pancreatic cancer [38]. Additionally, studies have demonstrated that the presence of CAFs can promote the proliferation of cancer cells in co-culture. A study by Gao et al. investigated the effects of CAFs on the proliferation of glioma cells in co-culture. The study showed that the presence of CAFs increased the proliferation of glioma cells through the secretion of growth factors such as FGF2 and interleukin-6 (IL-6) [39]. Similarly, a study by Sun et al. investigated the role of CAFs in promoting the survival and progression of breast cancer cells. The study finds that CAFs activate the FGF2/FGFR1 signaling pathway, which leads to the induction of autophagy and epithelial-mesenchymal transition (EMT) in breast cancer cells [40]. Overall, the increase in the amount of gold in the co-culture model could open the door for using GNPs in RT to exploit radiation-resistant co-culture systems.

### 2.4. DNA Double-Strand Breaks (DSBs) in Monoculture and Co-Culture

**Figure 5:**
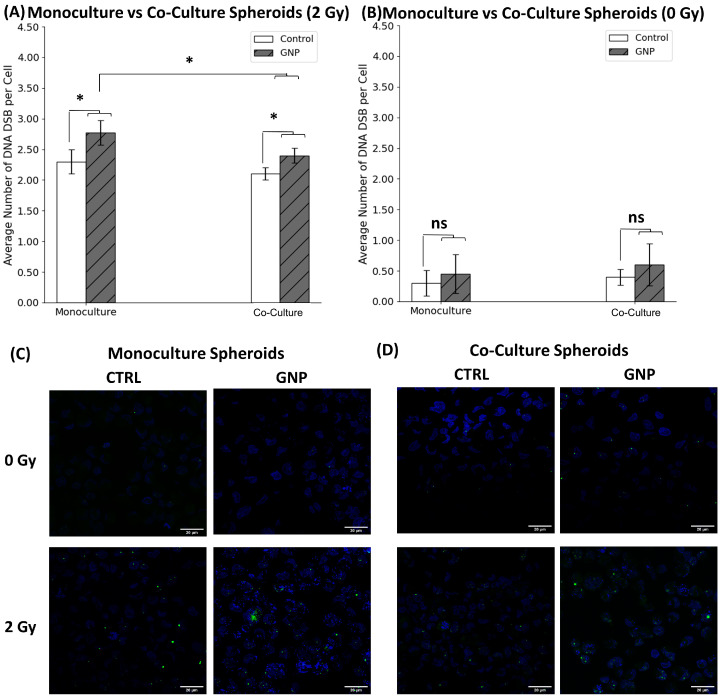
2D monoculture vs. 2D co-culture DNA DSB mapping. (**A**,**B**) The average number of DNA DSB per cell in 2D monoculture (**A**) and 2D co-culture (**B**) with and without radiation following treatments with different agents. Error bars showing standard error (S.E.), ns indicates non-significance, * indicates *p* < 0.05. (**C**,**D**) Confocal microscopy images of repair protein 53BP1 in the nucleus of monoculture MIA PaCa-2 (**C**) and in co-culture of MIA PaCa-2 and CAF-98 (**D**). The cell nuclei are stained blue, while the green dots indicate DNA DSB damage. Scale bar: 20 µm.

To further study the effects of radiation on our co-culture, an immunofluorescence assay was conducted to assess DNA damage. 53BP1 is a protein that is important for DNA damage response and repair. It plays a critical role in maintaining genome stability by recognizing and repairing DNA DSBs. Therefore, increases in this repair protein indicate greater DNA damage and DSBs. The 53BP1 foci were measured 24 h post-treatment and the average number of DNA DSB foci per cell for different conditions can be seen in Figure 5A with radiation and Figure 5B with no radiation. Confocal images of monoculture and co-culture samples highlighting 53BP1 foci in green and the nuclei in blue can be seen in Figure 5C,D. With RT/GNPs we see a significant increase in DNA DSB in both monoculture and co-culture (Figure 5A). GNPs with radiation resulted in 20.1% and 14.3% increase in DNA DSB per cell in monoculture and co-culture, respectively. Furthermore, co-culture cells demonstrated a significant reduction of 13.0% in DNA DSB damage when compared to monoculture cells subjected to RT/GNPs. With no radiation, no significant difference for GNPs relative to control was observed for either monoculture or co-culture (Figure 5B). These results are consistent with the mechanism of action of GNPs and the increase in resistance in co-culture systems as described earlier. When exposed to ionizing radiation, GNPs strongly absorb X-rays, resulting in increased cascades of Auger electrons leading to the production of free radicals and ROS. ROS are highly reactive molecules that can cause damage to the DNA in the form of DSBs inducing cell death even in more resistant co-culture systems.

## 3. Materials and Methods

### 3.1. Gold Nanoparticle Synthesis

Gold nanoparticles (GNPs) of an approximate size of 13 nm were synthesized using the citrate reduction method. It is a common and well-established technique to produce GNPs with small size distribution and good stability [41]. In this method, citric acid acts as both a reducing and stabilizing agent. The citrate ions reduce the tetrachloroaurate (AuCl4-) to gold nanoparticles, thus preventing their aggregation [41]. The process is carried out under mild conditions, typically at room temperature and atmospheric pressure. The citrate reduction method is economical and can yield GNPs of various sizes ranging from 2–150 nm, depending on the concentration of the citrate ions used [41]. The size and shape of GNPs produced using the citrate reduction method tend to be quite uniform due to the presence of citrate ions, which stabilizes the nanoparticles and prevents them from agglomeration. The process can be controlled by adjusting the temperature, the size of the gold precursor, the amounts of reactants, and the reaction time. GNPs images (Figure S2A) were taken using Transmission Electron Microscopy (TEM) (Ultra-high Resolution Scanning Electron Microscope SU9000, Hitachi, Pleasanton, CA, USA).

### 3.2. Gold Nanoparticle Functionalization

Two important functional groups used for surface modification of GNPs are polyethylene glycol (PEG) and arginine-glycine-aspartic acid (RGD). PEG, 2000 Da, is a hydrophilic polymer that has been used to modify the surface of GNPs to enhance their biocompatibility and stability [42]. The attachment of PEG molecules to the surface of GNPs reduces their tendency for aggregation and opsonization by the immune system [43]. PEGylated GNPs have shown improved biocompatibility, longer circulation times, and reduced toxicity, making them ideal for drug delivery applications [44]. RGD, 1600 Da, is a tripeptide sequence that specifically binds to the αvβ3 integrin receptor, which is overexpressed in several types of cancer cells and tumor vasculature including human pancreatic cancer cells [45]. Surface modification of GNPs with RGD peptides has been shown to significantly improve their targeting specificity towards cancer cells [46]. GNPs were functionalized with PEG and RGD at a surface density of 1 PEG per nm^2^ of the nanoparticle surface area and 1 RGD molecule for every 2 PEG molecules.

### 3.3. Gold Nanoparticles Characterization

The Perkin Elmer λ 365 UV-Vis spectrophotometer (Waltham, MA, USA) was used to measure the absorbance and transmittance of light to determine the size of the nanoparticles. UV-Vis spectrophotometry is a widely employed method for determining the size of GNPs. The extinction spectrum of gold nanoparticles displays one or more surface plasmon resonance bands, which were used to estimate the size of the nanoparticles with and without PEG, and RGD, as shown in Figure S2B and summarized in Figure S2E. ζ potential and dynamic light scattering (DLS) were used to characterize our functionalized and non-functionalized nanoparticles (LiteSizer 500 particle size analyzer, Anton Paar, Graz, Austria). These techniques provide information about the surface charge and size of the nanoparticles (shown in Figure S2C,D, and summarized in Figure S2E), which are important factors for their biological performance and stability. ζ potential is a measurement of the electrostatic charge around the nanoparticle’s surface. DLS, on the other hand, measures the size distribution of nanoparticles in solution.

### 3.4. Cell Cultures and Spheroid Formation

In this study, MIA PaCa-2 human pancreatic cancer cell line (ATCC#: CRL-1420™) were obtained from the American Type Culture Collection. Human pancreatic cancer-associated fibroblasts (CAF-98) were derived from a consenting patient’s resected pancreatic tumor tissue through the Gastrointestinal (GI) Biobank at Vancouver General Hospital. All cells were cultured in high glucose Dulbecco’s Modified Eagle’s Medium (DMEM; Gibco, ThermoFisher Scientific, Waltham, MA, USA) supplemented with 10% fetal bovine serum (FBS; Gibco, ThermoFisher Scientific, Waltham, MA, USA), 1% penicillin/streptomycin (Gibco, ThermoFisher Scientific, Waltham, MA, USA), and 4 mM of GlutaMax (Gibco, ThermoFisher Scientific, Waltham, MA, USA). Trypsin-EDTA (Gibco, ThermoFisher Scientific, Waltham, MA, USA) was used for cell detachment and paraformaldehyde (PFA; Sigma Aldrich, Oakville, ON, Canada) for cell fixation. Phosphate-buffered saline (PBS) was used for cell washing. Cell incubations were conducted at 37 °C with 5% CO_2_. Cells were seeded at a 5:1 ratio of CAF-98 to MIA PaCa-2 and incubated for three days for the 2D co-culture before initiating the experiments. For 3D spheroid cell cultures, cells were plated in ultra-low attachment 96-well microplates (Corning, NY, USA), with 6000 cells per well for MIA PaCa-2 and 1800 cells per well for monocultures with CAF-98 for a spheroid size of ~300–400 µm. The media was supplemented with 3% Geltrex matrix (Gibco, ThermoFisher Scientific, Waltham, MA, USA) on ice to help with spheroid formation. For co-culture spheroids, 300 MIA PaCa-2 and 1500 CAF-98 cells were seeded per well. Cells were centrifuged at 350× *g* for 5 min at 4 °C and incubated at 37 °C with 5% CO_2_. Experiments were initiated once the spheroids formed, following a 3-day incubation period.

### 3.5. Cellular Uptake of Gold Nanoparticle

GNPs were dosed following spheroid formation and the samples were incubated for 24 h. GNPs were dosed at a low concentration of 7.5 μg/mL. The samples were then washed with PBS five times and were incubated at 37 °C in trypsin-EDTA (Gibco, ThermoFisher Scientific, Waltham, MA, USA) for 1 h to help with breaking down the spheroids. Cells were then counted manually using a hemocytometer. Next, the samples were diluted in 5 mL Millipore water and treated with 250 μL of aqua regia for every 500 μL of the sample. The samples were placed in a 90 °C mineral oil bath for about 2 h. Subsequently, 100 μL of hydrogen peroxide was added to each sample, before putting them in the oil bath for 1 h. Thereafter, the samples were diluted with deionized water to a 2.5% *v*/*v* acid content. Finally, Inductively Coupled Plasma–Mass Spectrometry (ICP-MS; Agilent 8800 Triple Quadrupole, Agilent Technologies, Santa Clara, CA, USA) was used to measure the gold content in each sample in parts per billion (ppb) or ng/mL The calculation of gold nanoparticles per cell was performed using the following equation:

$$\frac{Gold\;nanoparticle\;}{Cell}=\frac{\frac{Gold\;Concentration}{Sample}\left[\frac{g}{mL}\right]\times Sample\;Volume\left[mL\right]\times N_{A}[\frac{atoms}{mol}]}{Gold\;atomic\;mass\left[\frac{g}{mol}\right]\times Number\;of\;Cells\times \frac{Gold\;atoms}{Gold\;nanoparticle}}$$

where $the\;atomic\;mass\;of\;gold=196.96657\frac{g}{mol},$ $N_{A}$ is Avogadro’s number ($6.022\times 10^{23}\frac{atoms}{mol}$), and the number of gold atoms per gold nanoparticle is measured using the following equation:

$$\frac{Gold\;atoms}{Gold\;nanoparticle}=\frac{Atoms\;per\;unit\;cell\times Gold\;Nanoparticle\;Volume\left[nm^{3}\right]}{Unit\;cell\;Volume\left[nm^{3}\right]}=\frac{4\times \frac{4\pi r^{3}}{3}}{a^{3}}=\frac{2}{3}\pi {\left(\frac{D}{a}\right)}^{3}$$

The calculation of the number of gold nanoparticles per cell was based on the core diameter of a spherical gold nanoparticle (D = 13 nm) and the length of a unit cell (a = 0.408 nm). Gold nanoparticles synthesized through the citrate reduction method adopt a face-centered cubic crystal structure with four gold atoms in each unit cell. We made two assumptions for this calculation: first, that the distribution of nanoparticles within each cell type is uniform, and second, that the size of gold nanoparticles is consistent throughout. These assumptions were supported by the images obtained from confocal microscopy for GNPs’ distribution and transmission electron microscopy (TEM) for GNPs’ size. The calculations represent an average for the entire group.

### 3.6. Gold Nanoparticle Imaging

Live 3D samples were imaged using a 20X lens of the Confocal microscopy (Zeiss LSM 980, Carl Zeiss Microscopy GmbH, Jena, Germany) to visualize GNPs distribution in the spheroids. After 3D spheroids were formed as described in Section 3.4 and dosed with GNPs as described in Section 3.5, they were transferred to 35 mm coverslip-bottom dishes (MatTek, Ashland, MA, USA) with just a few drops of media so that they did not dry out, and to ensure that they did not move during the imaging process. To visualize the gold, GNPs were conjugated with Cy5 fluorescent dye molecules. The Cy5 dye absorbs light in the range of ~600–700 nm and emits fluorescence in the range of ~650–750 nm.

### 3.7. Radiation Treatment

24 h following cell dosing with GNPs, and prior to radiation treatment, most of the media was removed carefully, samples were washed with PBS five times, and the media was changed. The cell-culture plates were placed between two 5 cm solid water blocks at the isocenter of a Varian TrueBeam linear accelerator (Palo Alto, CA, USA) at BC Cancer-Victoria in British Columbia, Canada, and irradiated with 2 Gy of radiation by a single beam incident from below. Then, 0 Gy control samples were transported to the linear accelerator to assure identical transportation conditions, but they were not irradiated. Other control samples were not dosed with GNPs or drugs but were irradiated at 2 Gy. The phantom thickness is chosen to ensure a uniform dose to all samples and with enough material to provide a full backscatter dose allowing for accurate dosimetry. All samples were then transported back to the lab to be processed for proliferation assay and Immunofluorescence assay to assess the efficacy of each treatment.

### 3.8. Cell Proliferation Assay and Spheroid Size

Following the radiation treatment, cell proliferation assay was conducted for 3D spheroids at days 1 and 14 post-treatment. Media was removed from each well, leaving only 100 µL of media, and then 30 µL of CellTiter-Glo 3D (Promega, Madison, WI, USA) was added to each well. Following a 0.5 h-incubation period, fluorescence was measured using Biotek Cytation 1 plate reader (Agilent Technologies, Santa Clara, CA, USA). For the size of the spheroids, following the radiation treatment, spheroid brightfield images were taken every three days using the 4× objective Biotek Cytation 1 plate reader (Agilent Technologies, Santa Clara, CA, USA). Manual assessment with the help of ImageJ was used to calculate the average diameter of the spheroids.

### 3.9. Immunofluorescence Assay

Monoculture and co-culture cells were incubated in 6-well dishes on glass coverslips. Then, 24 h following the radiation treatment, the samples were rinsed with PBS, and then fixed with 4% PFA for 5 min. After being fixed with PFA, the cells were rinsed with PBS, then washed with 2% BSA/0.1% Triton-X and incubated for 20 min. DNA DSBs damage was assessed using an optically labeled antibody against the repair protein, 53BP1. The 53BP1 primary antibody was diluted 1:200 in 0.5% BSA/0.1% Triton-X/PBS, whereas the secondary antibody was diluted 1:500 in 0.5% BSA/0.1% Triton-X/PBS. The samples were initially incubated with the primary antibody for 1 h, then rinsed with PBS. After that, the samples were washed with 0.5% BSA/0.175% Tween-20/PBS for 5 min and incubated with the secondary antibody for 30 min in the dark. The samples were then washed with PBS and mounted onto glass coverslips using ProLong™ Glass Antifade Mountant with NucBlue™ Stain (Invitrogen, Waltham, MA, USA). A 60× oil immersion lens was used to perform imaging of the 53BP1 foci through confocal microscopy (Zeiss LSM 980, Carl Zeiss AG, Jena, Germany). A minimum of 50 nuclei were assessed, and the number of foci per cell was measured.

### 3.10. Statistical Analysis

The Python (version 3.11.4) package statannot was used for conducting a statistical analysis through Welch’s *t*-test. The significance level was denoted with * for *p* < 0.05, ** for *p* < 0.01, and *** for *p* < 0.001. The experiments were conducted in triplicate, and the error bars indicate one standard deviation from the mean of the three measurements.

## 4. Conclusions

Pancreatic cancer is a deadly form of cancer with a poor prognosis. Although there has been progress made in its treatment, surgeries are not feasible for all patients and chemotherapy is not always effective and can result in severe adverse effects. Gold nanoparticles (GNPs) have emerged as promising research agents. Functionalized GNPs can increase the effectiveness of radiation treatment for cancer by locally depositing the energy from radiation and damaging cancer cells more effectively, while sparing healthy surrounding tissue. This study employed an in vitro 3D co-culture spheroid model made of MIA PaCa-2 and patient-derived cancer-associated fibroblasts (CAFs) of pancreatic origin to test the effectiveness of clinically relevant doses of GNPs (7.5 μg/mL)/RT (2 Gy). The paper highlights the advantages of using 3D co-culture spheroid models in testing cancer drugs in vitro providing an advanced platform for optimizing a treatment plan that can better mimic the in vivo tumor microenvironment (TME) easing the translation into clinical trials. This work showcased the therapeutic value of using GNPs with RT compared to using RT alone for the more resistant co-culture model. This combination showed a significant decrease in tumor size and cell proliferation and a significant increase in DNA DSB. This combination increases the therapeutic efficacy of RT by enhancing the sensitivity of tumor cells to radiation without inducing toxicity. While the combination of GNPs with RT shows promise in preclinical studies, there are some hurdles to overcome before they can be translated into clinical practice. The heterogeneity of tumors poses a significant challenge. Developing strategies to enhance tumor-specific accumulation and minimize off-target effects will be critical. Tumor cells can have diverse genetic backgrounds and expression patterns of specific receptors. Tailoring treatment strategies to individual patients based on their tumor characteristics may be necessary to maximize therapeutic efficacy. Considering their low toxicity, we are confident that the utilization of GNPs in combination with RT has the potential to enhance the efficacy of cancer treatment, leading to a higher likelihood of achieving successful remission and improved overall survival rates.

## Data Availability

Not applicable.

## Supplementary Materials

The following supporting information can be downloaded at: https://www.mdpi.com/article/10.3390/ijms241512523/s1.
