# Supplementary material for: Utilizing Gold Nanoparticles as Prospective Radiosensitizers in 3D Radioresistant Pancreatic Co-Culture Model
Source: Int J Mol Sci. 2023 Aug 7;24(15):12523. doi: 10.3390/ijms241512523 (PMC10419973; doi:10.3390/ijms241512523)
Supplement: Supplementary file 1 [file ijms-24-12523-s001.zip › ijms-2539963-supplementary.pdf]

Utilizing Gold Nanoparticles as Prospective Radiosensitizers in 3D Radioresistant Pancreatic Co-Culture Model

Abdulaziz Alhussan [1], Nolan Jackson [1], Reinali Calisin [1], Jessica Morgan [2,3], Wayne Beckham [1,4] and Devika B. Chithrani [1,4,5,6,7,*]

1 Department of Physics and Astronomy, University of Victoria, Victoria, BC V8P 5C2, Canada; alhussan@uvic.ca (A.A.); nolanjackson12@uvic.ca (N.J.); rcalisin@uvic.ca (R.C.); wbeckham@bccancer.bc.ca (W.B.)
2 Department of Biochemistry and Microbiology, University of Victoria, Victoria, BC V8P 5C2, Canada; jemorgan@bccrc.ca
3 Trev and Joyce Deeley Research Centre, British Columbia Cancer-Victoria, Victoria, BC V8R 6V5, Canada
4 Radiation Oncology, British Columbia Cancer-Victoria, Victoria, BC V8R 6V5, Canada
5 Centre for Advanced Materials and Related Technologies, Department of Chemistry, University of Victoria, Victoria, BC V8P 5C2, Canada
6 Department of Medical Sciences, University of Victoria, Victoria, BC V8P 5C2, Canada
7 Department of Computer Science, Mathematics, Physics and Statistics, Okanagan Campus, University of British Columbia, Kelowna, BC V1V 1V7, Canada
* Correspondence: devikac@uvic.ca

Supplementary Section S1: Monoculture Spheroids

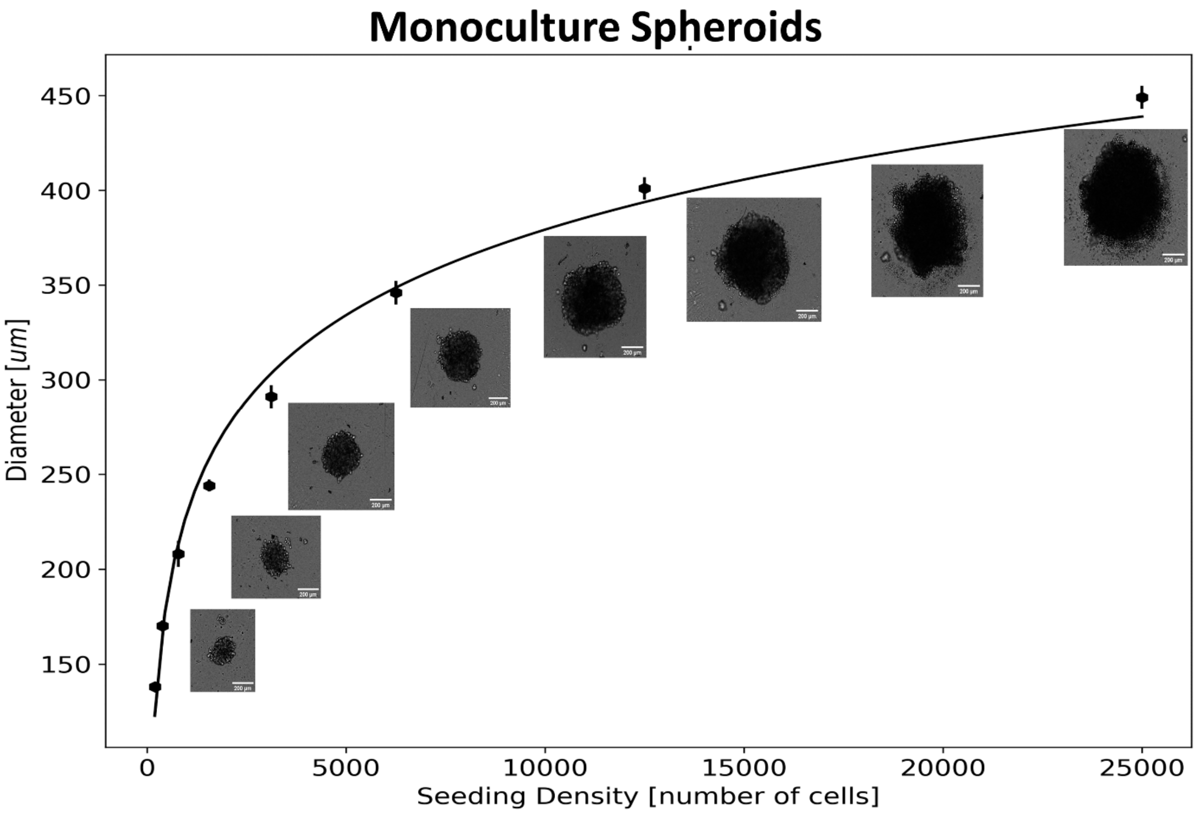

Figure S1. Characterizing pancreatic cancer 3D spheroid size. The size of the spheroids for MIA PaCa-2 monoculture under different initial cell count seeding. The scale bar is 200 μm.

Supplementary Section S2: GNPs Characterization

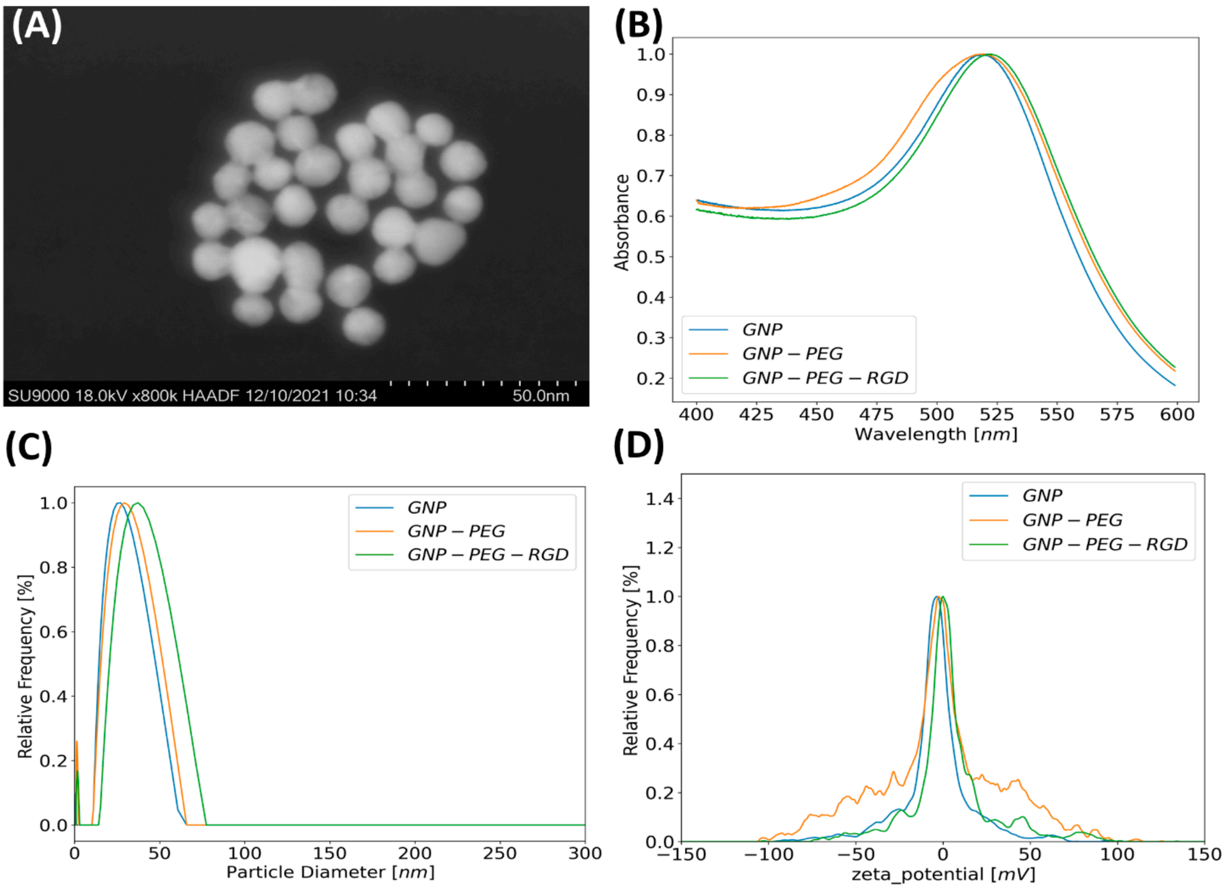

(E)

	Peak Wavelength (nm)	Hydrodynamic Diameter (nm)	Zeta Potential (mV)
GNP	519.15 ± 0.05	14.30 ± 1.46	−13.22 ± 2.89
GNP+PEG	520.05 ± 0.05	20.82 ± 2.01	−3.61 ± 1.22
GNP+PEG+RGD	524.35 ± 0.05	28.44 ± 3.79	0.82 ± 0.82

Figure S2. Gold nanoparticles (GNPs) characterization. **(A)** High-Angle Annular Darkfield (HAADF) Scanning Transmission Electron Microscopy (STEM) image of GNPs **(B)** UV–visible absorption spectra of GNPs, GNP$_{PEG}$, and GNP$_{PEG\text{-}RGD}$. **(C)** The hydrodynamic diameter of GNPs, GNP$_{PEG}$, and GNP$_{PEG\text{-}RGD}$. **(D)** The ζ-potential of GNPs, GNP$_{PEG}$, and GNP$_{PEG\text{-}RGD}$**. (E)** A table outlines the peak absorption wavelength, hydrodynamic diameter, and mean ζ-potential for GNPs, GNP$_{PEG}$, and GNP$_{PEG\text{-}RGD}$.

Supplementary Section S3: Spheroids Proliferation

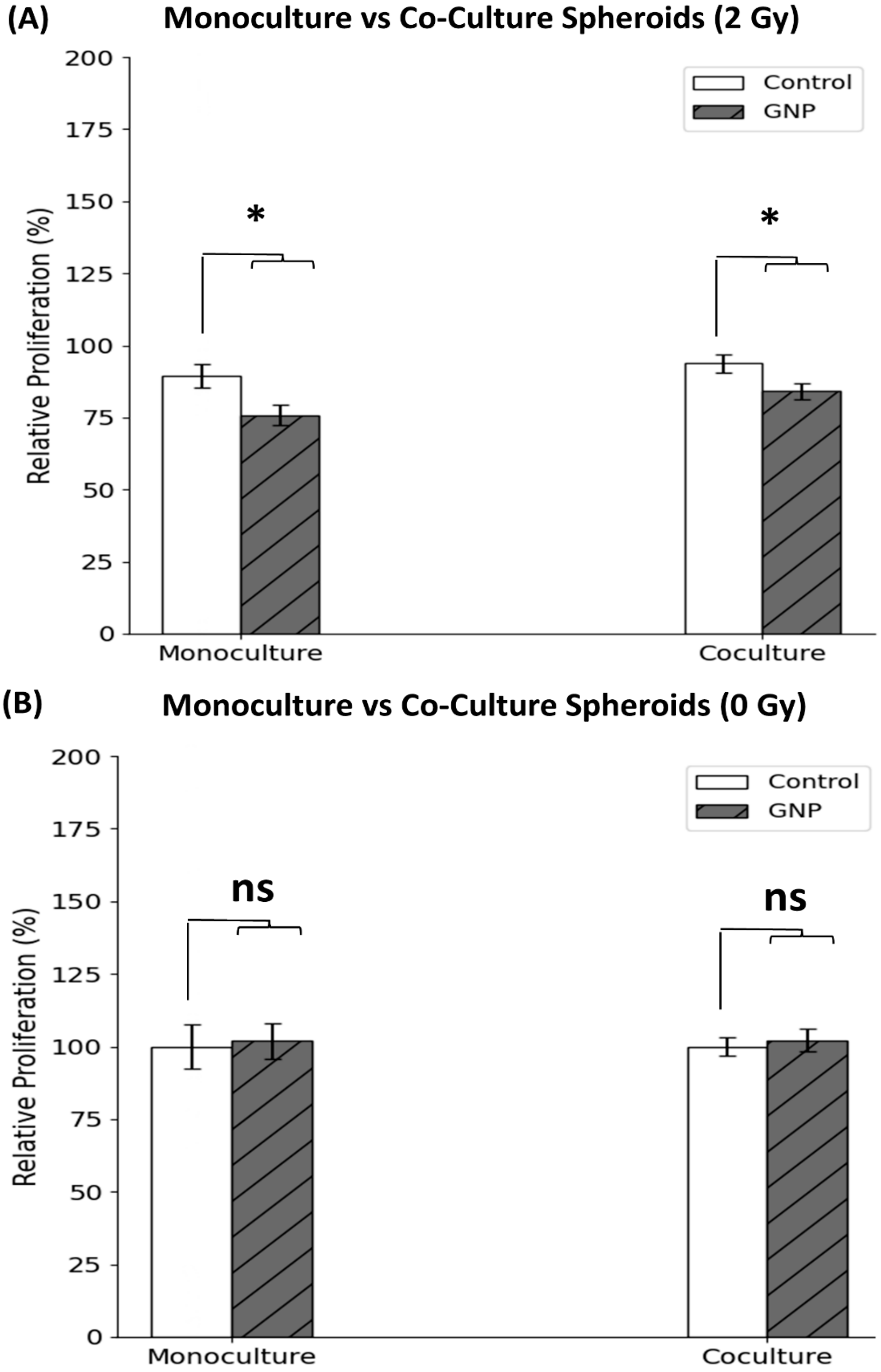

Figure S3. Monoculture vs co-culture spheroids proliferation post-treatment with radiation. **(A-B)** Irradiated spheroids **(A)** and non-irradiated spheroids **(B)** relative cell proliferation at day 14 post-treatment. ns indicates non-significance, * indicates $p < 0.05$.
